# Supplementary material for: An EGCG Derivative in Combination with Nimotuzumab for the Treatment of Wild-Type EGFR NSCLC
Source: Int J Mol Sci. 2023 Sep 13;24(18):14012. doi: 10.3390/ijms241814012 (PMC10531337; doi:10.3390/ijms241814012)
Supplement: Supplementary file 1 [file ijms-24-14012-s001.zip › ijms-2591086-supplementary.pdf]

General

Mass spectrometry (MS) data were obtained in the ESI mode on an API Qstar Pulsar instrument. ^{1}H-NMR and ^{13}C-NMR spectra were recorded on Bruker AVANCE III 500 MHz (Bruker BioSpin GmbH, Rheinstetten, Germany) instruments using tetramethylsilane (TMS) as an internal standard. Chemical shifts (δ) are presented in ppm and coupling constants (J) in Hz. Column chromatography (CC) was performed over a silica gel (200–300 mesh; Qingdao Makall Group Co., Ltd; Qingdao; China). All reactions were monitored via thin-layer chromatography (TLC), which was visualized using ultraviolet light (254 nm) and sprayed with 5% H_2SO_4 in EtOH, followed by heating. All reagents were commercially available and used without further purification unless indicated otherwise.

Experimental

Oxidation of EGCG, isolation and characteristics of EGCG oxides 1–2

To a solution of EGCG (200 mg) in 10 mL distilled water, potassium hexacyanoferrate (300 mg) and sodium hydrogen carbonate (100 mg) were added. The reaction mixture was stirred at 25°C, pH 7, for 15 min. Saturated citric acid solution was added and pH adjusted to 2, followed by extraction with ethyl acetate (3 × 10 mL). The combined organic phases were dried over anhydrous Na_2SO_4, filtered and concentrated under reduced pressure. The residue was purified via column chromatography with $CHCl_3/CH_3OH/AcOH$ (2:1:0.5%), leading to generation of compound **1** (6.4 mg, 3.2 %) as a yellow powder. The water phases were freeze-dried under vacuum and the resulting residue further purified via preparative HPLC with CH_3OH-H_2O (45%) to generate compound **2** (11.2 mg, 5.6%) as a yellow powder. Compound **1** was isolated as a yellow amorphous powder with negative ESIMS at m/z 913 $[M-H]^-$. ^{1}H-NMR and ^{13}C-NMR spectra of compound **1** corresponded to data reported in the literature (1). Compound 2 was assigned a molecular formula of $C_{44}H_{34}O_{23}$, determined via negative ESIMS at m/z 929 $[M-H]^-$, as well as NMR data, which displayed a signal pattern similar to dehydrotheasinensin A (2).

(2R,2'R,3R,3'R)-5,5',7,7'-tetrahydroxy-2,2'-bis(3,4,5-trihydroxyphenyl)-[4,8'-bichromane]-3,3'-diyl bis(3,4,5-trihydroxybenzoate) ***(Compound 1)***: Yield: 3.2%,

yellow powder; ^{1}H-NMR (CD_3OD, 500 MHz,) δ 6.91 (s, 2H, Ar-H), 6.74 (s, 4H, Ar-H), 5.91 (d, 1H, J = 2.3 Hz, $C^{6''}$-H), 5.88 (d, 1H, C^6-H), 5.87 (s, 1H, C^8-H), 5.25–5.23 (m, 3H), 4.90 (s, 1H, C^3-H), 4.72 (s, 2H, C^4-CH_2), 3.31-3.29 (m, 2H, $C^{4''}$-CH_2); ^{13}C-NMR (CD_3OD, 125 MHz) δ 167.8 (C=O), 157.7, 146.9, 146.3, 139.8, 129.5, 121.4, 112.3, 110.3, 108.3, 99.0, 96.6, 95.9, 76.3, 69.0, 26.9 (C-4), 20.8 (C-4''); ESIMS *m/z* 913 $[M-H]^-$.

(3R,3'R)-((2R,6S)-2,9,10,11,11-pentahydroxy-3-oxo-3,6-dihydro-2H-2,6-methanobenzo[b]oxocine-5,7-diyl)bis(5,7-dihydroxychromane-2,3-diyl) bis(3,4,5-trihydroxybenzoate) ***(Compound 2)*****:** Yield: 5.6%, yellow powder; ^{1}H-NMR (500 MHz, CD_3OD) δ 6.95 (s, 2H, Ar-H), 6.42 (s, 1H, $C^{2''}$-H), 5.96 (s, 1H, $C^{6'}$-H), 5.83 (d, 1H, J = 2.3 Hz, C^6-H), 5.90 (s, 1H, J = 2.3 Hz, C^8-H), 5.80 (d, 1H, J = 2.3 Hz, $C^{6'''}$-H), 5.75 (d, 1H, J = 2.3 Hz, $C^{8'''}$-H), 5.45 (s, 1H, $C^{2'''}$-H), 4.57 (s, 1H, $C^{3'''}$-H), 4.47 (s, 2H), 3.34 (s, 1H, $C^{2'}$-H), 3.16-3.12 (m, 2H, C^4-CH_2), 2.89-2.82 (m, 2H, $C^{4'''}$-CH_2); ^{13}C-NMR (CD_3OD, 125 MHz) δ 200.3, 167.7, 157.7, 157.0, 156.5, 140.1, 148.5, 148.0, 146.3, 139.8, 130.9, 128.0, 124.0, 121.1, 114.9, 110.2, 109.2, 99.7, 99.2, 96.8, 96.6, 95.8, 95.7, 94.0, 78.5, 69.3, 66.4, 54.9, 27.1, 27.0; ESIMS m/z 929 $[M-H]^-$.

Oxidation of EGCG, isolation and characteristics of EGCG oxide 3

To a solution of EGCG (200 mg) in 10 mL of 30% dioxane-water, $CuSO_4{\bullet}5H_2O$ (1 eq.) was added and the reaction mixture stirred at room temperature for 4 h. The mixture was concentrated until removal of dioxane and the residue purified via column chromatography with $CHCl_3/CH_3OH$/AcOH (2:1:0.5%), generating compound **3** (21.8 mg, 10.9 %). Compound 3 was isolated as an yellow amorphous powder. The negative-ion ESIMS at m/z 913 $[M-H]^-$ and NMR spectra of 3 corresponded to theasinensins A (3).

(2R,2'R,3R,3'R)-(4,4',5,5',6,6'-hexahydroxy-[1,1'-biphenyl]-2,2'-diyl)bis(5,7-dihydroxychromane-2,3-diyl) bis(3,4,5-trihydroxybenzoate) ***(Compound 3, theasinensins A)*****:** Yield: 10.9%, yellow amorphous powder; ^{1}H-NMR (500 MHz, CD_3OD) δ 6.92 (s, 4H, Ar-H), 6.75 (s, 2H, Ar-H), 5.91 (d, 2H, J = 2.9 Hz), 5.88 (d, 2H, J = 2.9 Hz), 5.28-5.27 (m, 2H), 4.73 (m, 2H), 2.79-2.75 (m, 2H), 2.54-2.49 (m, 2H); ^{13}C-NMR (CD_3OD, 125 MHz) δ 167.8 (C=O), 157.8, 157.7, 157.3, 146.9, 146.3, 145.4,

139.8, 134.4, 129.5, 121.4, 112.3, 110.3, 108.3, 99.0, 96.6, 95.9, 76.3, 69.0, 26.9 (C-4); ESIMS m/z 913 $[M - H]^-$.

Oxidation of EGCG, isolation and characteristics of EGCG oxide 4

A solution of EGCG (206 mg) in 10 mL phosphate buffer (Na_2HPO_4, 0.05 M) was incubated at 60°C for 2 h. The reaction mixture was extracted with ethyl acetate (3 × 10 mL) and the combined organic layer dried over Na_2SO_4. The solvent was evaporated under vacuum to obtain a residue, which was purified via column chromatography with $CHCl_3/CH_3OH$/AcOH (4:1:0.1%), yielding compound **4** (185 mg, 90%) as a white amorphous powder. The negative-ion ESIMS at m/z 457 $[M - H]^-$ indicates that compound 4 is an EGCG analog. NMR spectra support a *cis*-relationship between H-2 and H-3. This product was identified as GCG (4).

*(2S,3R)-5,7-dihydroxy-2-(3,4,5-trihydroxyphenyl)chroman-3-yl 3,4,5-trihydroxybenzoate (**Compound 4)*****:** Yield: 90%, white amorphous powder; ^{1}H-NMR (CD_3OD, 500 MHz) 6.93 (s, 2H), 6.48 (s, 2H), 5.93 (s, 2H), 5.52 (d, 1H, J = 2.4 Hz), 4.96 (d, 1H, J = 2.0 Hz), 2.59 (s, 1H, C^4-H_a), 2.51 (dd, 1H, J = 2.0 Hz, 3.6 Hz, C^4-H_b); ^{13}C-NMR (CD_3OD, 150 MHz) 167.6 (C=O), 157.9 (C-5, C-7), 146.7 (C-3', C-5'), 139.7 (C-4''), 133.8 (C-4'), 130.7 (C-1'), 121.4 (C-1''), 110.2 (C-2'', C-6''), 106.8 (C-2', C-6'), 99.4 (C-10), 96.8 (C-6), 95.8 (C-5), 78.6 (C-2), 69.9 (C-3), 26.8 (C-4); ESIMS m/z 457 $[M - H]^-$.

Table S1. Molecular interactions between theasinensins A and EGFR

RUN	Molecular Interactions	Nature of Interactions	Distance (Å)
1	LYS-237 ---- Lig	Hydrophobic interaction	2.4
2	LEU-245 ---- Lig	Hydrophobic interaction	3.7
3	ASP-238: O ---- Lig: H	Hydrogen bond	1.8
4	ASP-238: O ---- Lig: H	Hydrogen bond	1.9
5	THR-238: H ---- Lig: O	Hydrogen bond	3.3
6	LYS-229: H ---- Lig: O	Hydrogen bond	2.5
7	ASN-256: H ---- Lig: O	Hydrogen bond	2.6
8	GLU-259: O ---- Lig: H	Hydrogen bond	1.9
9	GLU-259: O ---- Lig: H	Hydrogen bond	2.2
10	THR-239: H ---- Lig: O	Hydrogen bond	2.7
11	ASP-238: H ---- Lig: O	Hydrogen bond	2.5
12	LYS-237: H ---- Lig: O	Hydrogen bond	2.1

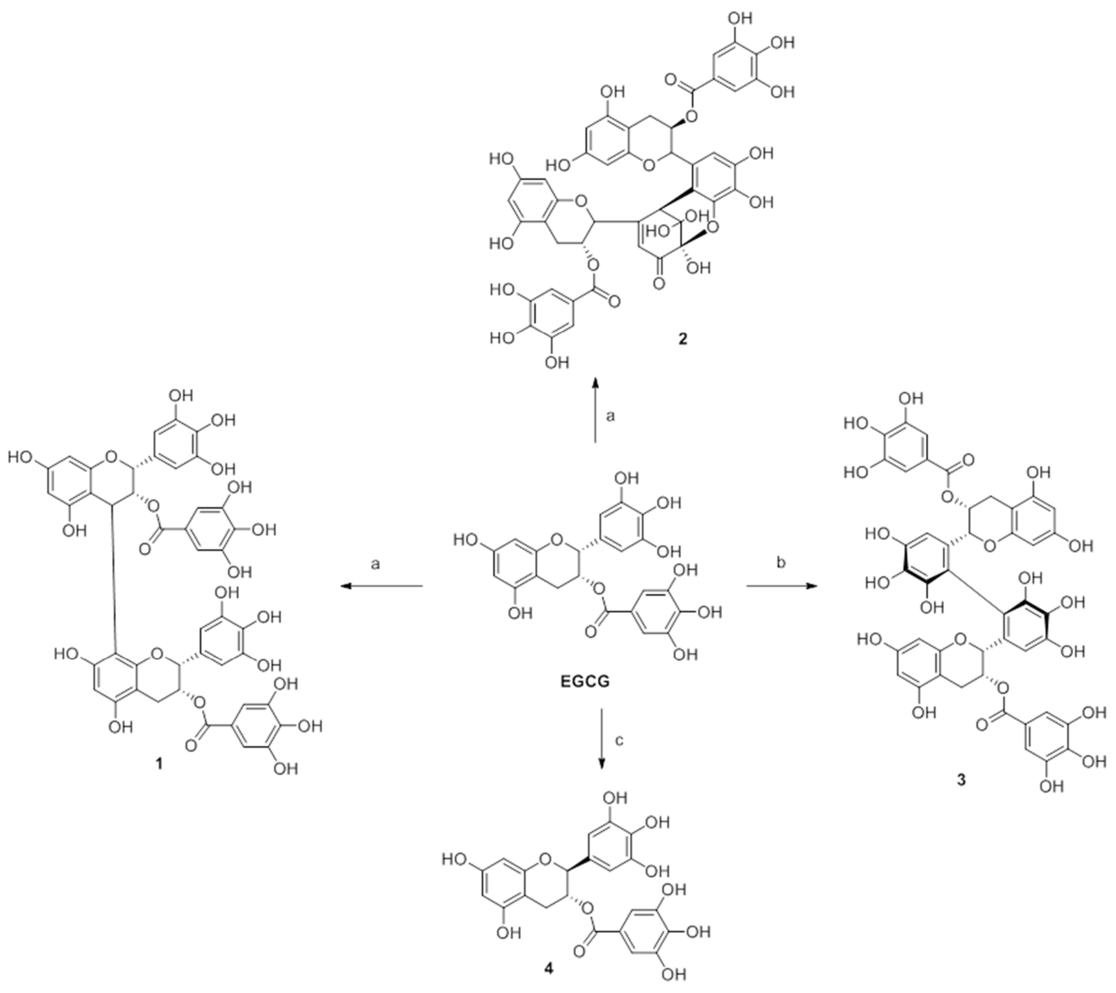

Figure S1. Synthesis of EGCG oxides (compounds **1**-**4**)

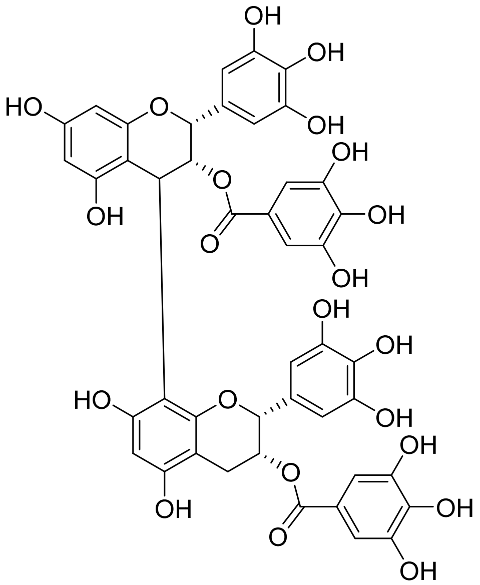

1

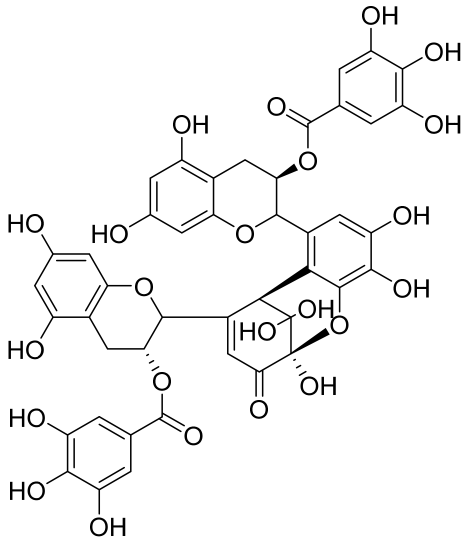

2

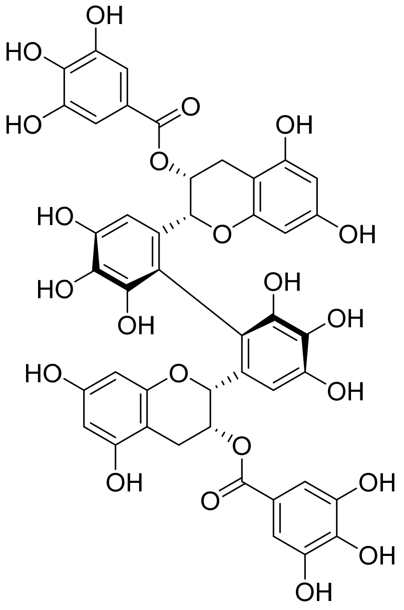

3

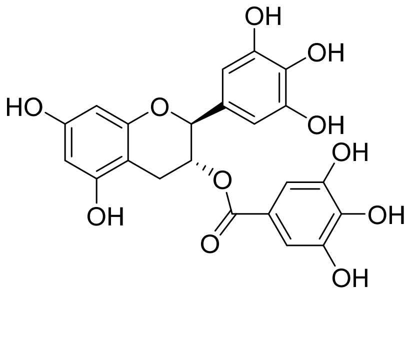

4

Figure S2. Chemical structures of EGCG oxides (compounds **1**–**4**)

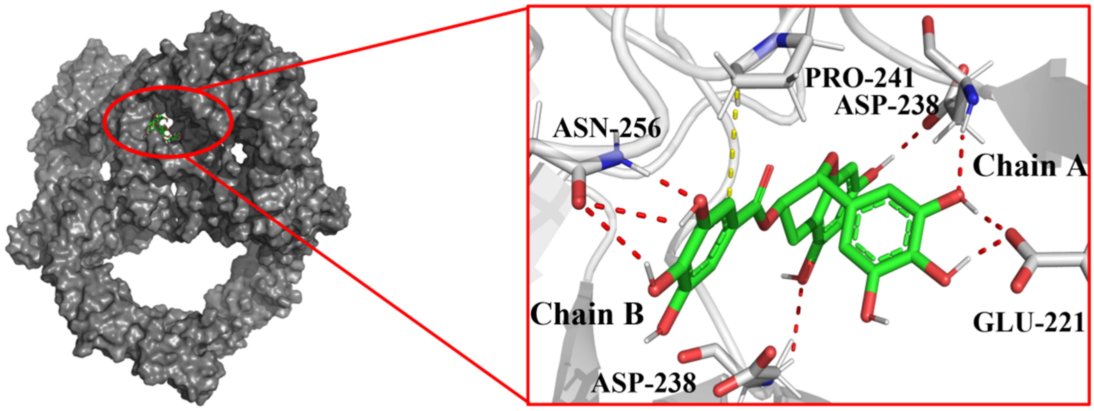

Figure S3. The binding models of EGCG and EGFR. The protein is shown as cartoon; ligand and the key residues are shown as cartoon sticks (ligand color: C green, N blue, O red and polar hydrogen); hydrogen bonds are shown as red dotted lines; hydrophobic interactions are shown as yellow dotted lines.

References

1. Wang Y, Wang J, Yang H, Zhang B, Zhang P, Sun P, et al. The oxidation of (-)-epigallocatechin-3-gallate inhibits T-cell acute lymphoblastic leukemia cell line HPB-ALL via the regulation of Notch1 expression. RSC Advances 2020;10(3): 1679-84.
2. Tanaka T, Watarumi S, Matsuo Y, Kamei M, Kouno. Production of theasine- nsins A and D, epigallocatechin gallate dimers of black tea, by oxidation-reduction dismutation of dehydrotheasinensin A. Tetrahedron 2003;59(40):7939–47.
3. Kashiwada Y, Nonaka G, Nishioka I. Tannins and related compounds. XXIII. rhubarb (4): isolation and structures of new classes of gallotannins. Chem Pharm Bull 1984; 32(9):3461–70.
4. Xie L, Guo Y, Cai B, Yang JJ. Epimerization of epigallocatechin gallate to gallocatechin gallate and its anti-diabetic activity. Med Chem Res 2013;22:3372–78.
